# Spermatogonial stem cell survival in ram lambs following busulfan treatment

**DOI:** 10.1590/1984-3143-AR2020-0001

**Published:** 2020-07-07

**Authors:** Mohammad Hadi Rasouli, Mohammad Zandi, Ali Asghar Sadeghi, Naser Emamjomeh-Kashan

**Affiliations:** 1 Department of Animal Science, Faculty of Agriculture and Natural Resources, Science and Research Branch, Islamic Azad University, Tehran, Iran; 2 Department of Agriculture, Iranian Research Organization for Science and Technology, Tehran, Iran

**Keywords:** spermatogonial stem cells, busulfan, recipients, sheep

## Abstract

To clarify the effect of busulfan on the depletion of spermatogonial stem cells (SSCs) from shal rams testis, in the first experiment, lambs were treated by intraperitoneal injection with 4 mg/kg busulfan. In the second experiment, different concentrations of busulfan (1, 2 and 4 mg/kg) were injected directly into both sides of the left testis. The testes of 8 lambs were collected by standard castration procedure for histological analysis five weeks after the treatments and the left testis of remaining lambs were collected after eight weeks and a two-time enzymatic digestion process was used to isolate SSCs. The results showed that all rams that had received intraperitoneal injections of busulfan died. But by testicular injecting of same dose of the drug, 40% of the animals died. The testicular injection of rams with 1, 2 and 4 mg/kg of busulfan resulted in a dose dependent decrease in testis size and also spermatocytes population after 5 weeks of treatments. From the results of colony formation 8 weeks after treatment with busulfan, it can be concluded that only in 1 and 2 mg/kg of busulfan, recovery of endogenous germ cells was performed. In conclusion, the results demonstrated that intra-testicular injections of busulfan (2 mg/kg) reduced spermatocytes population in ram testis within 5 weeks of treatments, and this effect was reversible within 8 weeks of injection. However, it was not recommended to inject 4 mg/kg busulfan into the peritoneal cavity or testis of lambs based on its side effects.

## Introduction

Transplantation of male animal germ cells is particularly important because it is used to produce transgenic animals, propagation of elite sires, and conservation of genetic resources (Herrid et al., 2009). Using this technology in sheep industry is of great importance due to the problems associated with sperm cryopreservation, artificial insemination, and embryo transfer, which are used as a tool for transporting and disseminating genetics (Rodriguez-Sosa et al., 2006).

The successful transplantation of spermatogonial stem cells (SSCs) largely depends on the preparation of the recipients, which destroys and suppresses internal germ cells and spermatogenesis, thereby enabling the replacement of transplanted SSCs and beginning donor-derived spermatogenesis (Ma et al., 2011). Various methods, such as genetically infertile hosts, chemotherapy, and radiation treatments, are used to eliminate host germ cells, leading to the improving the colonization of SSCs in the recipients (Lin et al., 2017).

Although SSC transplantation was practical to many nonrodent animals, SSC derived offspring were born only in goat, sheep, and chicken (Honaramooz et al., 2003; Trefil et al., 2006; Herrid et al., 2009; Takashima and Shinohara, 2018). Stockwell et al. (2013) showed the successful transplantation of isolated SSCs from rams into the irradiated testes of recipient rams and the production of offspring from transplanted cells. Rodriguez-Sosa et al. (2006) showed that, in seven of eight testes injected, donor cells were identified within the epithelium of the seminiferous tubule for up to 2 weeks after transplantation, representing the integration of donor cells. Recently, Zeng et al. (2013) have reported green fluorescent protein (GFP) expression in boars up to 64 months after transplantation. Stockwell et al. (2013) also reported the persistence of donor derived sperm in 12 recipient rams for up to 5 years post transplantation.

Although, the irradiation method was developed and has shown some hopeful results in rams; but this approach needs specialized and expensive instruments, and leads to calcification in the seminiferous tubules and this can impair the ability of the transplanted cells to flow through the seminiferous tubules (Ma et al., 2011).

A unique approach often used to prepare the recipients is the intraperitoneal injection of busulfan (1,4-butanediol dimethane sulfonate), which is easy to access and to use. Busulfan can be represented as a DNA alkylating agent, which is used to kill endogenous SSCs. Such a phenomenon results in emptiness in the basal and adluminal portions of seminiferous tubules before SSC is transplanted. Cytotoxic effects are caused by Busuflan when DNA-DNA cross-links, DNA protein cross-links, and single-strand breaks are formed. The toxi effect of busulfan can be exerted on cells in the G0/G1 phase of the cell cycle (Ganguli et al., 2016).

Busulfan has been used to prepare SSCs transplant recipients in a variety of species, including mouse, rat, monkey, and pig (Ogawa et al., 2003; Zhang et al., 2003; Hermann et al., 2007; Honaramooz et al., 2005). But intraperitoneal injection of busulfan in rodents can inhibit hematopoiesis and severe side effects and sometimes cause recipients’ death (Qin et al., 2016a).

In larger animals such as pigs, and smaller animals such as mouse and rats, busulfan-based treatment can cause systemic toxicity and even death as a result of severe bone marrow destruction (Ma et al., 2011). In young and adult male rats, high sensitivity to the toxic effects of busulfan results in incomplete removal of germ cells and compromised testicular health (Honaramooz et al., 2005). The side effects of busulfan treatment significantly reduce the efficacy of transplantation of SCCs and affect the welfare of recipient animals, especially when the recipients are endangered animals or livestock species (Ma et al., 2011). Although busulfan injection can be used to prepare recipient in non-roden species, the timing and dosage appropriate for each species should be optimized (Kubota and Brinster, 2018). In this study, injection of busulfan directly into the testis was compared with intraperitoneal injection in order to provide a low-cost, feasible, and safe preparation method.

## Methods

### Chemicals

Unless otherwise stated, purchasing all chemicals was done from Sigma (St. Louis, MO, USA), and plastics were bought from Sorfa (China).

### Experiment

Shal male lambs at 4^th^ months of age were purchased from Rasoli Farm (Tehran, Iran) and the protocol for animal use in the present investigation was approved by the Iranian Research Organization for Science and Technology (IROST) Agricultural Institute of Animal Ethics, Care and Use. Male lambs were randomly assigned into two experiments - three lambs in the first experiment and twenty lambs in the second experiment (five lams in each group of second experiment), which ensure similar scrotal sizes and body weights in each group. In the first experiment, the lambs were injected intraperitoneal with 4 mg/kg doses of the dissolved busulfan in 40 mg/mL dimethyl sulfoxide (DMSO). In the second experiment, the direct injection of 1, 2 and 4 mg/kg doses of the dissolved busulfan in 40 mg/mL DMSO into both sides of the scrotums of left testis was conducted equally by using 5-mL syringes, as described by Lin et al., 2017. The 0 mg/kg group was considered as the control group, and the dose of the injected DMSO was the same as the experimental groups. Five weeks after the treatments the testes of 8 lambs were collected by standard castration procedure for histological analysis. Eight weeks after the treatment, the left testis of the remaining lambs were collected and transferred to laboratory within 2 hours after castration for isolation and culture of SSCs.

### Histology

Pieces of tissue from testes were fixed overnight at 4 °C in modified Davidson’s fluid (Latendresse et al., 2002). They were subsequently washed in 70% ethanol, embedded in paraffin and sectioned at 5 mm using standard procedures. Sections were processed through xylene and ethanol into water and stained with hematoxylin and eosin for histological examination.

### Isolation and culture of SSCs

The left testis of the rams was collected 8 weeks after busulfan treatments. In order to isolate SSCs, the researchers benefited from a two-time enzymatic digestion process as described by Izadyar et al. (2003) with some modifications. Briefly, the removal of the tunica albuginea was conducted for the first enzymatic digestion. It is worth noting that cutting ~50 g of tissue into small pieces was carried out by fine scissors, and suspending minced seminiferous tissue was done in Dulbecco’s Modified Eagle Medium (DMEM)(Inoclon, Iran). It contains 1 mg/mL trypsin (Inoclon), 1 mg/mL hyaluronidase type II, 1 mg/mL collagenase, and 5 μg/mL DNase. Incubation was conducted at 37 °C in a shaker incubator (200 cycles/min) for 45 min. The collection of the dispersed tissue was done and the centrifugation was conducted at 1000 rpm for 2 min. The collection of supernatant was done and DMEM was used in order to wash the pellet. Regarding the second enzymatic digestion, the suspension of the pellet was conducted in DMEM, which contains 1 mg/mL hyaluronidase type II, 1 mg/mL collagenase, and 5 μg/mL DNase. Incubation was also done in a shaker incubator (200 cycles/min) for 30 min. The suspension was then centrifuged at 1000 rpm for 2 min.

### Enrichment of SSCs

The enrichment of SSCs was done through filtering the supernatant by a 80 µm and then a 60 µm nylon net filter. Then, transferring the filtered cells to lectin- bovine serum albumin (BSA), which coats 60 mm petri dishes, was done as described by Jafarnejad et al. (2018). The preparation of the lectin-BSA coated dishes was conducted through dissolving lectin (5 µg/mL) from Datura stramonium agglutinin in Dulbecco’s Phosphate-Buffered Saline (DPBS). Keeping the dishes at room temperature was done for 2 hours. After that, BSA was used for washing them (0.6% BSA in DPBS). For coating BSA, the dished were also held at room temperature for another 2 hours. The incubation of the seeded cells on the lectin-coated dishes was done for 5-6 hours at 37 °C in a CO_2_ incubator with 5% CO_2_ in air. Such an incubation process enables most of contaminating cells to get attached to the lectin-BSA. Then, the collection and the transfer of the remaining medium to 15 mL tube were conducted, while it was expected to contain SSCs. Its centrifugal action was done for 5 min at 1000 rpm. The supernatant was subsequently discarded and the re-suspension of the pellet in DMEM took place.

### Preparation of feeder layers

Fresh DMEM supplemented with 10% FBS (Gibco, Life Technologies, Rockville, MD, USA) was used to revitalize the leftover cells in the lectin-coated dishes. The incubation process was conducted in a CO_2_ incubator with 5% CO_2_ in air at 37 °C for 2-3 days. Incubation aimed to enable these cells, which were expected to be primarily sertoli cells, to grow till a confluent monolayer was formed. Given that propagation was concerned, sub-culturing the cells was done in 50 ml cell culture flask after being disaggregated with 0.25% trypsin-EDTA. In order to prepare a feeder layer, inactivation of sertoli cells was done by treatment with 10 μg/mL mitomycin-C for 3 hours. Washing the cells took place for 5 times with DPBS. Finally, they were washed with DMEM supplemented with 10% FBS.

### Culture of SSCs

Culturing the isolated SSCs was conducted on the sertoli cells feeder layer in 50 ml cell culture flasks containing DMEM medium supplemented with 10% FBS, and then incubated in a CO_2_ incubator with 5% CO_2_ in air at 37 °C. SSC colonies were observed in primary culture after 10 days.

### Characterization and analysis of SSCs

In order to characterize SSCs, Alkaline phosphatase staining and expression of *c-myc*, *plzf* and *gfra1* genes were used. As to the staining of the alkaline phosphatase, washing SSC colonies with DPBS were done twice. Then, it was stained using an alkaline phosphatase kit (Sigma, Catalogue No.86C) as the manufacturer’s protocol. The numbers of colonies were counted under inverted microscopy.

### RNA isolation, reverse transcription and real time PCR

The isolation of total RNA was conducted with Trizol reagent (Invitrogen Corp., Carlsbad, California, USA). After that, it was treated with DNAse (Ambion Inc., Houston, Texas, USA) to avoid DNA contamination. By measuring the absorbance at 260 nm, the concentration of extracted total RNA was achieved. A total of 0.5 mg of total RNA was used for the first-strand complementary DNA (cDNA) synthesis. Reverse transcription was done with MMLV enzyme and oligo dT primers (Takara, Japan). The changes in expression of specific markers were studied by real time-PCR. PCR was set up in a final volume of 10μL having 5µl sybergreen, 1.4 µl nucleaus free water, 0.8 µl each of forward, and reverse primers and 2 µl template. The real time-PCR program was started with an initial melting cycle at 94 °C for 15 min to activate the polymerase, followed by 40 amplification cycles of denaturation at 95 °C for 10 sec, annealing specific primers at 60 °C for 15 sec and 72 °C for a 20-sec extension. The reactions were ended with a final extension at 72 °C for 5 min. The following custom primer sequences were used for real time PCR gene expression analysis: β *actin* [5' ACCCAGCACGATGAAGATCA 3' (forward) and 5' GTAACGCAGCTAACAGTCCG 3' (reverse)]; *plzf* [5'CCTCAGATGACAATGACACG 3' (forward) and 5'CGCCTTGGTGGGACTCA 3' (reverse)]; *c-myc* [5' AGAATGACAAGAGGCGGACA 3' (forward) and 5' CAACTGTTCTCGCCTCTTC 3' (reverse); *gfra1* [5' CCACCAGCATGTCCAATGAC 3' (forward) and 5' GAGCATCCCATAGCTGTGCTT 3' (reverse)]. Comparative threshold cycle (∆∆CT) method was used in order to analyze the data, and *β actin* was employed as an endogenous control.

### Statistical analysis

A statistical software program (SPSS 16, IBM, USA) was run for data analysis. The inferential analyses of one-way ANOVA as well as Duncan multiple-range test were applied in order to compare multiple numeric datasets. The major results of the study were stated as mean±SEM and statistical significance was accepted at P <0.05.

## Results

### Busulfan side effects

In the first experiment, two days after intraperitoneal busulfan injection (4 mg/kg), two lambs died of diarrhea with lethargy and the other ones were injured on their mouth after 4 days and died after 20 days. In the second experiment, the testicular injection of 4 mg/kg busulfan killed two of the five lambs in this group.

### Testicular weights after busulfan treatment

**Figure 1:**
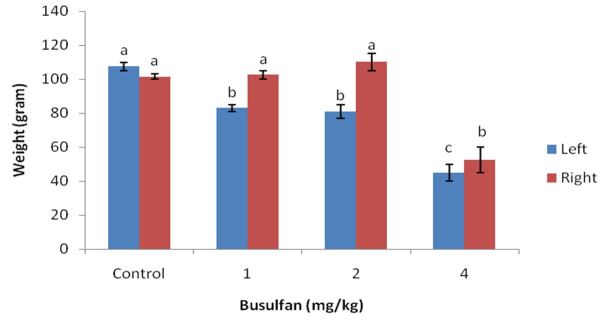
Mean testicular weight in control and busulfan (1, 2 and 4 mg/kg) treatments .

Figure 1 shows the testis weight of experimental rams after direct injection of busulfan (1, 2 and 4 mg/kg) to left testis. The left testicular weight was significantly lower in the busulfan group than in the control group (p<0.05). The testes weight in the 4 mg/kg busulfan group reached its lowest level, which was significantly lower than the average weight in the control group (p<0.05). No significant differences were observed between 1 and 2 mg/kg busulfan and control group in right testicular weight (p>0.05) (Figure 1).

### Histological analysis

**Figure 2:**
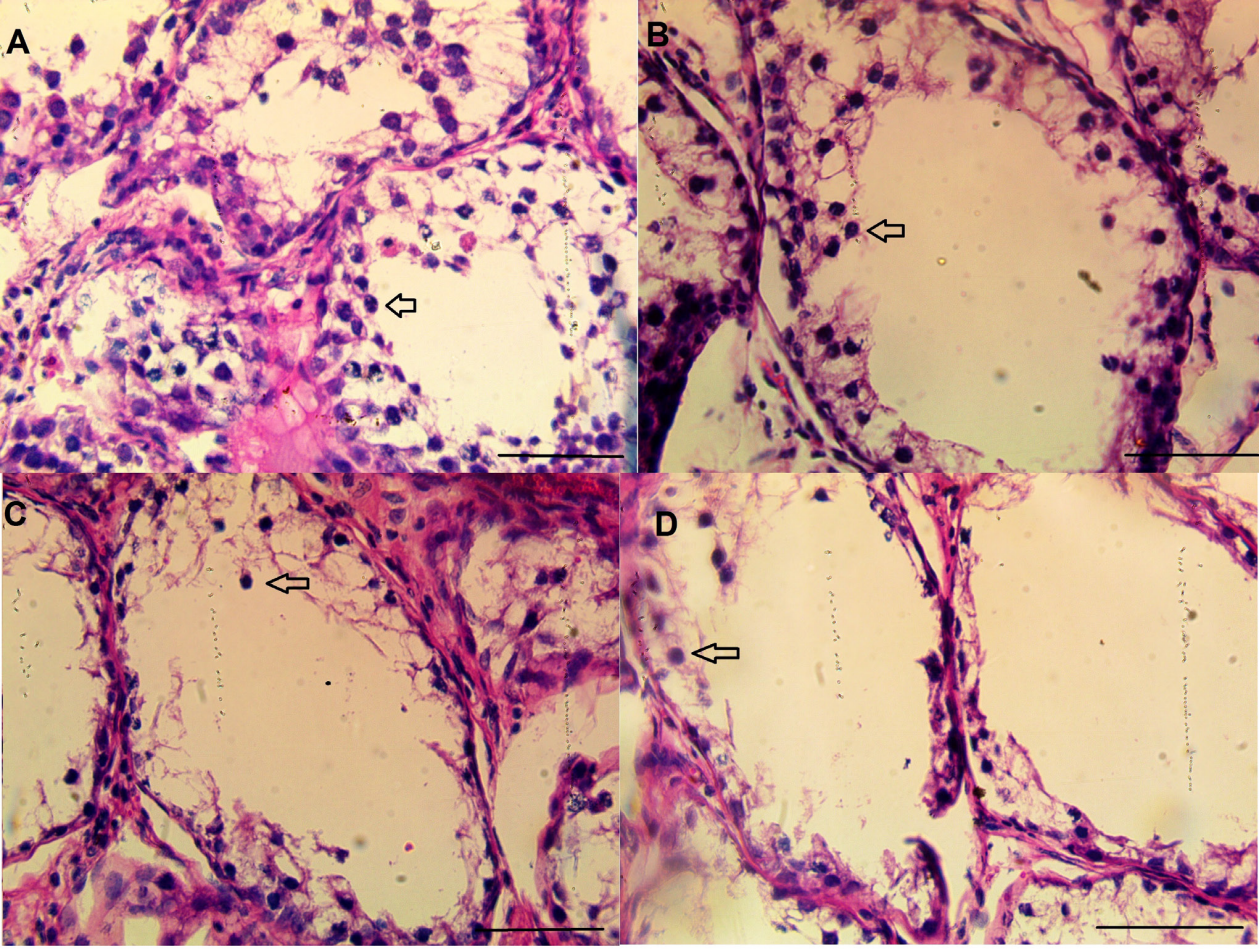
Histology of tissue from ram testes. Light micrograph of a section stained with hematoxylin and eosin. Spermatogenesis is not fully established with spermatocytes being the most advanced type of germ cell (arrows). The busulfan doses are 0 (A), 1 mg/kg (B), 2 mg/kg (C), and 4 mg/kg (D) (bars = 50 µm).

As shown in Figure 2, the treatment of shal rams with 1, 2 and 4 mg/kg of busulfan resulted in a dose dependent decrease in spermatocytes population on the basal lamina 5 weeks after the treatments.

### *In vitro* SSCs colony formation after 8 weeks of testicles busulfan treatments

**Figure 3:**
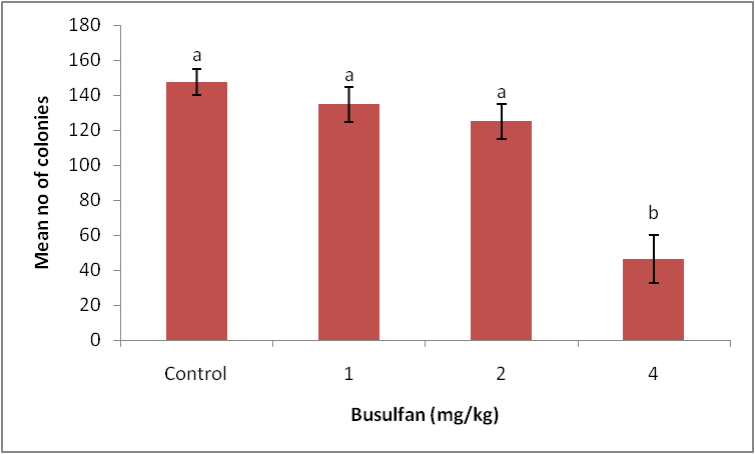
The effect of different concentrations of busulfan (1, 2 and 4 mg/kg) on the mean number of SSCs colonies.

**Figure 4:**
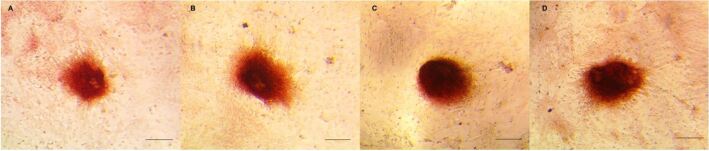
Alkaline phosphatase staining of ovine SSCs. The busulfan doses are 0 (A), 1 mg/kg (B), 2 mg/kg (C), and 4 mg/kg (D) (Bar = 0.5 mm). SSC colonies appeared after 7 days in culture and were observed after 10 days.

Ten days after *in vitro* culturing the SSCs isolated from the testicles treated with busulfan, results showed that 1 and 2 mg/kg busulfan did not show significant differences in colony formation with control, but 4 mg/kg busulfan significantly reduced the mean number of the formed colonies (Figure 3). SSC colonies showed alkaline phosphatase activity in all groups (Figure 4).

### SSCs markers gene expression

**Figure 5:**
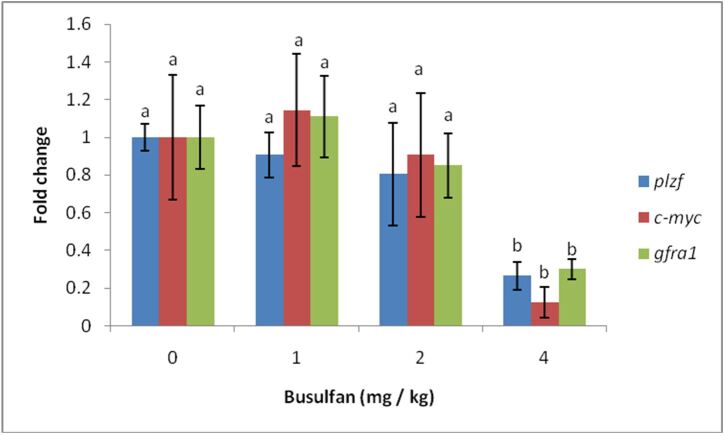
Real-time-PCR analysis of *plzf*, *c-myc* and *gfra1* genes of busulfan (1, 2 and 4 mg/kg) treated SSCs.

Results of the testicular injection of busulfan into the expression of *plzf*, *c-myc* and *gfra1* genes showed that 4 mg/kg busulfan significantly reduced the expression of these genes (p<0.05) and no significant difference were observed between 1 and 2 mg/kg of busulfan compared to the control group (p>0.05)(Figure 5).

## Discussion

Although germ cell transplantation is well under way in rodents, research in large animals has not sufficiently been advanced to prove the efficacy of this method in farm animals. One limiting factor in the development of this technology is the preparation of proper recipients (Ma et al., 2011). If transplant recipient testes are low or without SSCs, the colonization efficiency of spermatozoa can be improved as a result of germ cell transplantation. Busulfan, as a DNA alkylating agent that destroys dividing cells, has always been used to kill germ cells at transplant recipients prior to transplantation in rodents. But the effective dose of busulfan is species- and strain-specific, and busulfan-based treatment can be lethal due to severe bone marrow depression (Goharbakhsh et al., 2013).

To identify the appropriate dose of busulfan in ram, Olejnik et al. (2005) used systemic injection of busulfan (4, 8 and 16 mg/kg) for evaluating the side effects of this drug. They showed that doses of 8 mg/kg and above, which result in diarrhea with lethargy and lack of appetite after 5 days, are lethal to the survival of the animal (Olejnik et al., 2005). High mortality rates were also reported in pigs using 10-15 mg/kg of busulfan for 5 weeks (Honaramooz et al., 2005). Nevertheless, animals in the 4 mg/kg group showed only mild clinical effects that were not life-threatening (Olejnik et al., 2005). However, Olejnik et al. (2018) used a dose of 4 mg/kg in their experiment and reported a dramatic falls in white blood cells and platelets numbers, which were recovered within 3 weeks (Olejnik et al., 2018).

The results of our study showed that all rams that had received intraperitoneal injections of busulfan (4 mg/kg) died. But by testicular injecting of same dose of the drug, 40% of the animals died. The route of busulfan administration may affect its toxicity. There have been several reports of side effects of intraperitoneal injection of busulfan, causing systemic toxicity including bone marrow injury or animal death (Ganguli et al., 2016). In the study by Ganguli et al. (2016) no deaths were reported in busulfan-treated rats by intra-testicular injection, while approximately 60% of deaths were reported in the intraperitoneal injection of busulfan (Ganguli et al., 2016). Qin et al., 2016 also reported that intra-testicular injections of busulfan in mice reduced spermatogonia numbers although it caused less toxicity than intraperitoneal injections (Qin et al., 2016a).

The results of Olejnik et al. (2018) revealed that sheep are more sensitive to the toxic effects of busulfan compared to other species. While 4 mg/kg caused a small effect on spermatogonia, higher doses of it would carry a very high risk of animal health due to bone marrow toxicity (Olejnik et al., 2018). Susceptibility to busulfan varies even in pig breeds; however, different types of mice have been shown to be resistant to it (Ganguli et al., 2016; Qin et al., 2016b).

In our study, the testicular injection of shal rams with 1, 2 and 4 mg/kg of busulfan resulted in a dose dependent decrease in testis size and also spermatocytes population after 5 weeks of treatments. Systemic injection of busulfan reduced endogenous spermatogonia in the pre-pubertal ram (Olejnik et al., 2005), however, Olejnik et al. (2018) reported that spermatogonia numbers were not significantly reduced by using 4 mg/kg busulfan intravenously, although 27% were changed, indicating that the power of their experiment was insufficient to detect a difference (Olejnik et al., 2018).

Whereas our results showed that after 8 weeks of treatments no significant difference were observed among 1 or 2 mg/kg of busulfan group with control, based on SSCs colony formation and expressions of SSCs markers (*plzf* and *c-myc* genes), but by using 4 mg/kg busulfan, significant decrease was observed in SSCs colony formation and the expression of SSCs maker (p<0.05). From the results of colony formation 8 weeks after treatment with busulfan, it can be concluded that only in 1 and 2 mg/kg of busulfan, recovery of endogenous germ cells was performed. Lin et al. (2017) found the recovery of endogenous germ cells slightly after three months of busulfan treatment in Seghers pigs (recovery of Pgp9.5 mRNA level).

## Conclusion

To conclude, the results demonstrated that intra-testicular injections of busulfan (2 mg/kg) reduced spermatocytes population in ram testis within 5 weeks of treatments, and this effect was reversible within 8 weeks of injection. However, it was not recommended to inject 4 mg/kg busulfan into the peritoneal cavity or testis of lambs based on its side effects.
